# Promoting daily physical activity in Norway as a FYSAK school: a comparative longitudinal study of lower secondary school pupil

**DOI:** 10.3389/fspor.2025.1543741

**Published:** 2025-07-14

**Authors:** Ingeborg Barth Vedøy, Knut Ragnvald Skulberg, Patrick Foss Johansen, Hege Eikeland Tjomsland, Miranda Thurston

**Affiliations:** ^1^ Faculty of Health and Social Sciences, Inland Norway University of Applied Sciences, Elverum, Norway; ^2^ Department of Sport, Food and Natural Sciences, Western Norway University of Applied Sciences, Bergen, Hordaland, Norway

**Keywords:** physical activity, physical activity recommendations, FYSAK school, sustainability, equity, delivery

## Abstract

**Background:**

Schools have been described as “ideal settings” for promoting PA, but they have struggled to find effective and sustainable ways of doing so given the demands of the curriculum. In Norway, the FYSAK school model is an example of a population approach to PA promotion wherein daily PA is embedded into the routines of the school. Little is known about the extent to which the model supports pupils' daily PA, especially with regard to meeting the national recommendations. The paper addresses the following research question: what impact does the FYSAK model have on pupils' PA patterns over time?

**Methods:**

The paper draws on device-measured PA data from a three-year (2016–2018) longitudinal study of lower secondary school pupils from 11 schools in Norway, of which one was a FYSAK school. Data from 535 adolescents (56.1% female, mean age at baseline ± SD 13.3 ± 0.3 years) was derived and used to compare the PA level (cpm^−1^) of the one FYSAK school in the sample with the other 10 schools.

**Results:**

There was no difference in PA level between category of school in 2016. In 2017 and 2018 however, pupils attending the FYSAK school had significantly higher levels of PA compared to control schools (2017: 54.7 cpm^−1^, *p* ≤ .05, 2018: 59.2 cpm^−1^, *p* ≤ .05). Analyses of weekdays only, reinforced this pattern where larger differences in PA level across category of schools became evident (2017: 73.5 cpm^−1^, *p* ≤ .001, 2018: 85.7 cpm^−1^, *p* ≤ .001). Pupils attending the FYSAK school were also significantly more likely to adhere to the national recommendations for PA compared to control schools throughout all three years (2016: 57% FYSAK vs. 41% control, 2017: 62% FYSAK vs. 38% control, 2018: 52% FYSAK vs. 30% control).

**Conclusions:**

Overall, the results are indicative of a FYSAK school effect, which can be explained in terms of the sustained embedding of PA into pupils' daily routines over a three-year period. We conclude that the FYSAK model offers a framework for systematically providing realistic opportunities for being physically active during the school day.

## 1 Introduction

Evidence of the benefits of regular physical activity (PA) for young people's physical and mental health, as well as their academic achievement, has accumulated in recent years (1). The most recent guidelines from the World Health Organization (WHO) for school-aged young people (5‒17-years-old) recommend 60 min of moderate- to vigorous-intensity PA (MVPA) daily alongside limiting the amount of time spent being sedentary (2). While many countries (including Norway) have incorporated these recommendations into their PA guidance frameworks, few young people meet them (3). Schools are widely regarded as having a role in facilitating PA and thus supporting the achievement of such recommendations. They are, however, increasingly viewed as part of the problem in that contemporary patterns of schooling often give rise to extended periods of sedentary time—itself an independent risk factor for current and future health problems (4)—and limited opportunities for promoting PA in a crowded curriculum. While there has been a plethora of school-based initiatives (including time-limited interventions) over the past three decades or more, there is little evidence of their impact on either increasing PA during the school day (5) or on outcomes such as mental health and academic achievement (6, 7). The disadvantages of limited-duration interventions have, in particular, become widely recognized: promising results in the short-term are rarely maintained as schools struggle to sustain PA promotion post-intervention (8). Promoting PA by adapting everyday school routines may be a more effective and sustainable organizational model, yet it has rarely been researched (9). The departure point for this paper is, therefore, to explore the impact of such an organizational model of PA promotion—specifically the Norwegian school physical activity [FYSAK^1^] model. We first provide an overview of school-based PA promotion globally before focusing more specifically on the Norwegian context.

### 1.1 School-based PA promotion

Jourdan et al. (10) draw a distinction between “discrete programmatically based interventions […] and more systemic approaches that underpin sustainable health-promoting practices”. Although varied in structure, duration, and form, the field of school-based PA promotion has, to date, largely been characterised by the former, which have shown limited effectiveness and sustainability due to their typically time-bound nature and lack of wider integration (11–13). Systemic approaches—often referred to as whole school or whole-of-school approaches, draw, to varying degrees, on the WHO framework for health promoting schools originally established in the 1980s. Few countries, however, have successfully implemented such approaches (10) even though evidence suggests that whole school approaches can be effective in improving learning and health outcomes (14–16). Typically, PA promotion has taken the form of “tightly-constructed content” (8). However, given the well-documented limitations of such approaches, there has been some diversification in recent years. The integration of PA into curriculum subjects—physically active learning (PAL)—has been one such development (see, for example, 7, 17). Broader still, are whole-of-school multi-component approaches such as those that have emerged in the US, which aim to “implement comprehensive and coordinated approaches to PA to provide all students with opportunities to be physically active while on school grounds” (18). Such approaches have also developed in Europe (19).

### 1.2 School-based PA promotion in Norway

As far as school-aged children are concerned, recent device-measured PA studies indicate that Norway is one of the most physically active countries in Europe (20). Consistent with other countries, however, the proportion of girls and boys meeting the PA recommendations declines through the primary and secondary school years. For example, in 2018, 40% of 15 year-old girls and 51% of boys of the same age met the recommendations compared to 64% of girls and 81% of boys at age 9 (21). Thus, schools are viewed as important settings for countering these trends. The most recent public health White Paper articulates an ambition for schools to incorporate daily PA into their schedules (22) but the government has fallen short of mandating daily PA across all schools (23). Although this may be indicative of a reduced commitment to institutionalizing regular PA, schools in Norway have, over many years, responded to the ambition to promote PA, in a variety of ways: the use of extended breaks; the incorporation of PAL into lessons in a way that combines movement with academic content; interest-based physical education; and the FYSAK model, wherein a school schedules PA during the day as part of curriculum time without it being linked to any specific subject (24). Our interest is in the FYSAK school model. This model can be seen as a direct response to the requirements set forth in the Education Act §1–7, which mandates that all students have the right to regular physical activity outside of PE. Unlike other school-based PA initiatives that are often tied to specific subjects or learning objectives (e.g., PE or PAL), the FYSAK model is distinct in its primary aim to provide additional opportunities for movement without being explicitly linked to curricular goals. The flexibility of the FYSAK approach means that implementation varies between schools in terms of format, timing, and frequency. It typically entails breaking up the school day according to a planned schedule, with short periods of activity such as dancing or table tennis depending on a school's access to resources, so that pupils can accumulate an additional 30 min of PA during each school day. The overarching principle is to provide a low-threshold form of PA that does not require changing clothes. This makes it an accessible and inclusive solution, ensuring that all students, regardless of skill level or motivation, can participate regularly in physical activity as a normal part of their school day. Thus, the model aligns with the regulation's aim to ensure that students engage in PA beyond structured PE lessons, recognizing the broader benefits of physical activity for health, while also being widely assumed to support well-being and learning.

Our overall purpose is to investigate the impact of the FYSAK model on PA using an observational study design in a real-world setting. The paper thus addresses the following research question: what impact does the FYSAK model have on PA patterns over time?

## 2 Methods

### 2.1 Design and participants

This paper draws on data from a larger study (the schools, learning and mental health study) (25), which was designed to explore relationships between physical activity, mental health and academic achievement. This longitudinal cohort study of 13‒16-years-olds in Norway was conducted between 2016 and 2018. The participants were recruited from 11 lower secondary schools situated in two counties on the west and east side of Norway. Each school was recruited to the study via an initial visit that entailed the collection of qualitative information from the leadership, which provided insights into how PA initiatives, such as the FYSAK model, were implemented in the school environment. The schools were selected to provide a diverse sample that represented schools in Norway on the basis of geographical location, school size, type of school, socio-economic status of the school population and the school's average on standardized national tests. The participation rate for the entire study population was 59.8%, 58.0% and 55.1% for 2016, 2017 and 2018 respectively [for details, see (26)]. For Søndre Land specifically, the participation rate was 100% in all three years. In total, 621 participants generated data for at least one year; 441 participants provided valid PA data for two years or more. A detailed description of the methodology has been published elsewhere (25). This paper draws on data relating to patterns of PA over time. In particular, it compares the one FYSAK school in the sample, Søndre Land lower secondary school, with the other 10 schools.

### 2.2 Søndre land lower secondary school: a FYSAK school

Søndre Land is a large municipality in the Inland county of Norway. At the start of the study (2015‒ 2016), the municipality had one of the highest number of people (adults between 18‒49 years of age) on social welfare in Norway. Many pupils came from disadvantaged backgrounds, as indicated by the public health profile for the area, with relatively high levels of parents with low levels of education and income (27). Specifically, a higher percentage of children (0‒17 years) lived in low-income households compared to the national average, which highlighted the economic challenges faced by families in the region.

At the time of the study, Søndre Land was the only lower secondary school in the municipality and drew pupils from four primary schools. Because of this, many pupils travelled by bus to school. In 2015‒2016, there were 220 pupils in grades 8‒10 (age 13‒16 years) and 38 teachers. The school was located in an area of natural beauty close to Randsfjord and had good facilities, including access to a swimming pool and sports hall. It had been a FYSAK school since 2002, offering compulsory daily physical activity for all pupils. The PA initiative was led and co-ordinated by the headteacher with all teachers participating daily. The school leadership regarded FYSAK as having become a central part of the school's identity (see website^2^). Its work has been recognized nationally (24).

PA promotion has been embedded into the school's routines in order to ensure that pupils are active daily for 30 min (in addition to PE, which occurred 1–2 days a week). One of the PE teachers had responsibility for drawing up monthly FYSAK plans for all pupils. The pupil council also provided input into the organization and content of activities. Time for PA was made available by reallocating it from breaks, as well as by taking five minutes from all subjects over the course of each day. Activities were teacher-led, relatively simple, easily organized, and required no change of clothing; they included walking, dancing, orienteering, frisbee golf, skating, table tennis, corridor running, table bouldering (24). The initiative's goal was to seamlessly incorporate movement throughout the school day, with the hope of improving not only physical health but also mental wellbeing. The school also had a strong focus on outdoor life, easily facilitated given its location. This included a three-day trip in Jotunheimen mountains every autumn.

### 2.3 Measurements

#### 2.3.1 Physical activity

Physical activity was measured by accelerometry (ActiGraph GT3X+ and GT3X-bt, LLC, Pensacola, Florida, USA). Trained personnel attached the accelerometer on the participant's right hip at first wear and participants were instructed to wear the accelerometer for seven consecutive days. The accelerometer was only to be removed before sleep and water activities as it was not completely waterproof. PA data were included when participants had at least 4 days of valid measurements. A valid day consisted of ≥480 min of wear time. Intervals of ≥20 consecutive minutes of no recorded acceleration were defined as non-wear time. The accelerometer was initialized to start recording data the day after they were handed out to participants. Data recorded between 00:00 and 06:00 were excluded from the analysis. An epoch of 10 s was used to capture the potentially intermittent PA level of adolescents (28). Initialisation, downloading and processing of accelerometer data were conducted through ActiLife (v6. 13.4).

As a measure of the habitual PA level, average daily counts·min^−1^ (cpm) for the entire assessment period was used. Measuring habitual PA is recognized as being challenging because of the variation across days, weeks, and seasons (29). As there is currently no agreed approach to characterize habitual PA by accelerometer, one solution to increase stability of data is to create mean PA variables across the three years of data collection. Consequently, mean PA-variables were derived by including the mean PA value across a minimum of two waves of data collection. Participants with <2 PA measurements were excluded from these analyses. Analyses of the overall PA level included both weekdays and weekend days. To explore the specific PA level on school days, analyses including weekdays only was used (“PA level weekdays”). We defined sedentary behaviour (SED), light intensity PA (LPA) and moderate-to-vigorous PA (MVPA) as <100 cpm, 100–1,999 cpm and ≥2,000 cpm respectively. These cut-points have been used in previous studies of Norwegian school-aged young people's PA levels (30). Further, the Norwegian recommendation for PA “accumulation of an average of ≥60 min MVPA/day” was used to categorize participants into adhering/not adhering to national recommendations (31).

#### 2.3.2 Socio-economic status (SES)

The Family Affluence Scale version three (FAS III) was used to measure SES. The scale measures material affluence, which serves as a much-used proxy for SES (32). The scale consists of six questions reflecting material affluence (33). Based on these questions, a score of *relative family affluence* can be derived by summing scores on all answers and categorizing them into three broader groups (the lowest 20%, the middle 60% and the highest 20%).

#### 2.3.3 Transportation to/from school

Transportation to/from school was assessed through two questions where participants were asked about their usual travel mode to and from school. The category “Active transportation” consists of participants reporting either walking or biking to/from school. These questions have been used in previous studies of Norwegian school-aged young people's PA levels (30).

#### 2.3.4 Covariates

Sex and SES are identified as two of the most important covariates that confound the analysis of PA levels amongst school-aged young people (21, 34, 35). Consequently, the effect of both variables should be controlled for in analyses. However, due to the relatively small numbers of participants at Søndre Land school we were unable to control for both variables in the analyses. Given the well-documented evidence of differences in PA level related to sex (36), as well as the limitations of FAS III with regard to highly affluent study samples (32), we thus decided to adjust solely for sex in analyses of PA level.

### 2.4 Analyses

**Figure 1:**
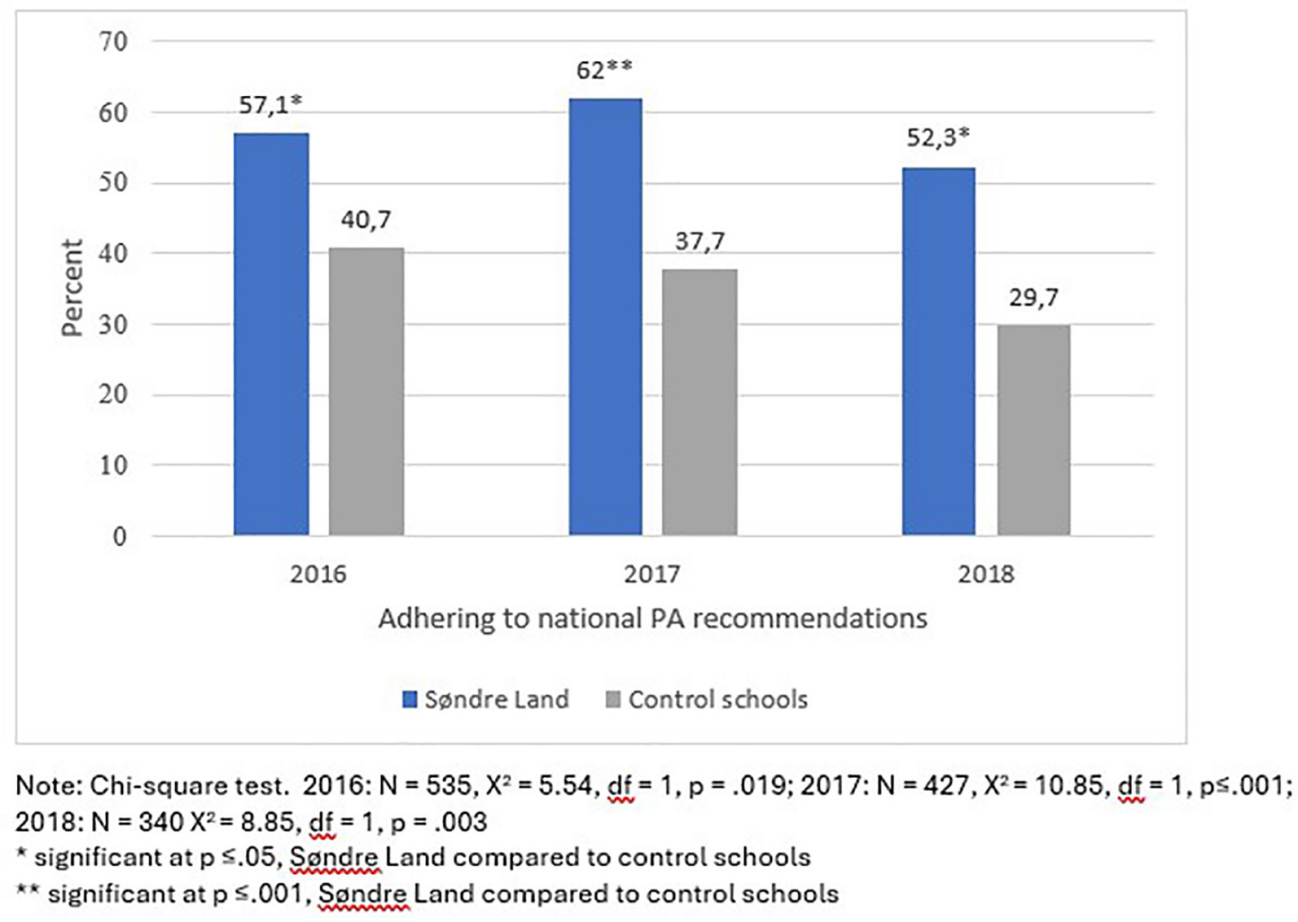
Percentage of Søndre, Land pupils compared to control school pupils adhering to national recommendations for PA in 2016, 2017 and 2018.

Statistical analyses were performed using IBM SPSS for Windows, Version 28.0.1.1. Descriptive data are presented as frequencies (n and percent) and mean (±SD) where appropriate (Table 1). To explore differences in descriptive characteristics between Søndre Land and control schools, Chi-Square tests were applied for the nominal variables, and Independent-samples *T*-test for the interval level variables. Analysis of covariance (Ancova) was used to explore differences in PA levels between Søndre Land and control schools adjusted for sex, both for overall PA level and PA level for weekdays only (Tables 2, 3). For the nominal variables, Chi-square tests were performed (Figure 1). Effect sizes attributable to adhering to national PA recommendations by category of school (Fysak vs. non-Fysak) were explored by Phi and Cramer's V. These analyses were not stratified by sex due to the low number of pupils at Søndre Land school.

**Table 1:** Descriptive characteristics of the study sample by school category, all time points (mean ± SD unless otherwise specified).

Characteristic	Time 1 (2016)		Time 2 (2017)		Time 3 (2018)	
	Søndre Land	Control schools	Søndre Land	Control schools	Søndre Land	Control schools
N	56	479	50	377	44	296
Boys (*n*, %)	31 (55.4)	204 (42.6)	26 (52.0)*	141 (37.4)	19 (43.2)	97 (32.8)
Girls (*n*, %)	25 (44.6)	275 (57.4)	24 (48.0)*	236 (62.6)	25 (56.8)	199 (67.2)
Age	13.3 (0.3)	13.4 (0.3)	14.3 (0.3)	14.4 (0.3)	15.3 (0.3)	15.4 (0.3)
SES						
Lowest 20% (*n*, %)	23 (41.8)**	99 (21.5)	11 (22)*	40 (11.5)	11 (26.2)*	33 (11.7)
Middle 60% (*n*, %)	30 (54.5)**	283 (61.4)	34 (70.8)*	240 (68.8)	29 (69.0)*	195 (69.4)
Highest 20% (*n*, %)	2 (3.8)**	79 (17.1)	3 (6.3)*	69 (19.8)	2 (4.8)*	53 (18.9)
Use of active transportation						
To school (*n*, %)	18 (31.6)**	314 (60.4)	21 (38.2)*	277 (57.5)	20 (37.7)*	266 (57.6)
From school (*n*, %)	18 (32.2)**	332 (64)	22 (40.0)*	289 (59.9)	19 (35.8)**	276 (59.8)
PA level (cpm/d)	433.9 (115.2)	408.7 (147.8)	463.1 (123.9)**	396.7 (148.2)	433.8 (149.2)*	367.1 (138.0)
SED (min/d)	582.8 (59.7)	593.2 (67.5)	571.4 (71.1)*	596.9 (74.2)	596.7 (77.4)	615.2 (76.0)
LPA (min/d)	173.9 (32.7)*	163.3 (33.6)	167.6 (25.5)*	156.8 (33.4)	146.5 (33.0)	137.0 (31.4)
MVPA (min/d)	62.8 (19.3)*	56.8 (20.7)	65.2 (17.8)**	54.8 (21.42)	62.4 (23.4)*	50.4 (20.5)
Adherence PArec (n,%)	32 (57.1)*	195 (40.7)	31 (62.0)**	142 (37.7)	23 (52.3)*	88 (29.7)

Note: Only data for participants with ≥4 days of valid PA measurements the same year are presented. (N differs depending on characteristics).

SES, socio economic status; BMI, body mass index; PA level (cpm/d), average daily counts per minute; SED, average daily sedentary behaviour; min/d, average minutes per day; LPA, average daily light intensity physical activity; MVPA, average daily moderate-to-vigorous intensity physical activity; Adherence PArec, adherence to national recommendations for PA.

* significant at *p* ≤ .05, Søndre Land compared to control schools.

** significant at *p* ≤ .001, Søndre Land compared to control schools.

**Table 2:** Analysis of covariance for the PA level (cpm/d) all days between Søndre Land and control schools with sex as a covariate.

Year	PA level (cpm/d)						
	Søndre Land		Control schools				
	Adjusted mean (SE)	N	Adjusted mean (SE)	N	F (df, error)	*P*-value*	Adjusted r square
2016	426.3 (18.9)	56	409.6 (6.5)	479	.694 (1, 532)	.405	.05
2017	452.6 (19.9)	50	398.1 (7.2)	377	6.60 (1, 424)	.011	.08
2018	430.1 (20.9)	44	367.6 (8.0)	296	7.77 (1, 337)	.006	.03
Mean 2016–2018	434.0 (17.3)	52	390.0 (6.3)	389	5.72 (1, 438)	.017	.08

Note: Only data for participants with ≥4 days of valid PA measurements the same year are presented. PA level (cpm/d), average daily counts per minute; df, degree of freedom; SE, standard error.

* *P*-values refer to differences in adjusted mean in PA level after controlling for differences among the groups with regard to sex.

**Table 3:** Analysis of covariance for the PA level (cpm/d) weekdays between Søndre Land and control schools with sex as a covariate.

Year	PA level (cpm/d)						
	Søndre Land		Control schools				
	Adjusted mean (SE)	N	Adjusted mean (SE)	N	F (df, error)	*P*-value*	Adjusted r square
2016	477.6 (20.9)	56	444.0 (7.1)	479	2.32 (1, 532)	.129	.05
2017	501.8 (20.1)	50	428.3 (7.3)	377	11.79 (1, 424)	≤.001	.09
2018	485.5 (21.7)	44	399.8 (8.34)	296	13.59 (1, 337)	≤.001	.07
Mean 2016–2018	486.5 (17.9)	52	421.9 (6.5)	389	11.51 (1, 438)	≤.001	.08

Note: Only data for participants with ≥4 days of valid PA measurements the same year are presented. PA level (cpm/d), average daily counts per minute; df, degree of freedom; SE, standard error.

* *P*-values refer to differences in adjusted mean in PA level after control for differences among the groups with regard to sex.

### 2.5 Ethics

The study was registered with the Norwegian Agency for Shared Services in Education and Research (Sikt) under project number 48192. Written informed assent from participants and consent from their legal guardians were obtained prior to data collection.

## 3 Results

### 3.1 Descriptive data

Descriptive data on key study variables are shown in Table 1. In 2017, there were significant differences in the proportion of girls and boys at Søndre Land school compared to control schools. Furthermore, there were systematic differences in the distribution of pupils in the low, middle and high SES categories across all three years. Analyses of school travel mode showed that a significantly lower proportion of Søndre Land pupils used active transportation to/from school compared to control schools across all three years. Further, analyses of intensity-specific PA showed that Søndre Land pupils had significantly more minutes in MVPA compared to control schools throughout the three years.

### 3.2 Physical activity level

There were no differences in PA level between Søndre Land and the control schools after controlling for sex in 2016 (Table 2). However, analyses of data from 2017–2018 showed that Søndre Land pupils had a significantly higher PA level than the control school pupils after controlling for sex. Beta values showed that the mean differences in PA level between Søndre Land and control schools increased during the three years (16.6 cpm^−1^, 54.7 cpm^−1^ and 59.2 cpm^−1^ higher PA level in Søndre Land in 2016, 2017 and 2018 respectively). There were also significant differences in PA level between boys and girls, where boys had 66.8 cpm^−1^, 73.1 cpm^−1^ and 33.8 cpm^−1^ higher mean PA levels than girls in 2016, 2017 and 2018 respectively (data not shown). The analysis of mean PA level (2016–2018) also showed that Søndre Land pupils had a significantly higher PA level than control school pupils. In this analysis, sex accounted for a somewhat larger difference in PA level (63.0 cpm^−1^) than category of school (44.0 cpm^−1^).

Analyses of PA level for weekdays only showed the same pattern of no significant differences between Søndre Land pupils and control school pupils in 2016 (mean difference 33.6 cpm^−1^, NS) (Table 3). For 2017 and 2018, Søndre Land pupils had a significantly higher PA level for weekdays than control school pupils (an increase of 73.5 cpm^−1^ and 85.7 cpm^−1^ compared to control schools in 2017 and 2018 respectively). Category of school introduced larger differences in PA level for weekdays compared with analyses of the PA level for the entire week (73.5 cpm^−1^ vs. 54.7 cpm^−1^ in 2017 and 85.7 cpm^−1^ vs. 59.2 cpm^−1^ in 2018). The analysis of mean PA level for weekdays (2016–2018) (Table 3) also showed that Søndre Land pupils had a significantly higher PA level than control school pupils (64.6 cpm^−1^), which was equal to the difference introduced by sex (63.2 cpm^−1^) and higher than the difference found in analysis of the mean PA level of the entire week (44.0 cpm^−1^).

### 3.3 Adherence to national PA recommendations

The proportion of pupils adhering to national recommendations for PA was significantly higher for Søndre Land pupils compared to control school pupils in all three years (Figure 1). The effect sizes attributable to adhering to national PA recommendations by type of school for the different years were small according to Cohen's interpretation of effect sizes for Cramer's V; 0.10, 0.16 and 0.16 respectively.

## 4 Discussion

This paper set out to explore the potential of the FYSAK school model for promoting daily PA compared to other schools over a three-year period. Our results showed that pupils at Søndre Land school had significantly higher levels of PA (both all days and weekdays) compared to control schools for 2017 and 2018. Although the differences in 2016 did not reach statistical significance, this might be accounted for in terms of data collection occurring early in the Autumn semester of the first year of lower secondary school before new routines had become established. The mean PA level for all three years (2016‒2018: both all days and weekdays), which is a stronger measure of the habitual PA level (29), was also significantly higher for Søndre Land school than for control schools. When we excluded weekend days from the PA measurements, the difference in PA level for Søndre Land compared to control schools was even greater. This gives further support to the higher levels of PA in Søndre Land school compared to control schools. We interpret these results in terms of the school strengthening its influence on pupil's PA level over time. Taken together these results are supportive of a FYSAK school effect.

Accordingly, pupils attending Søndre Land school were statistically more likely to meet the PA recommendations compared to those at control schools in all three years. Given the well-documented decline in PA levels during the teenage years with fewer meeting the recommendations (20, 21), this is a noteworthy finding. In particular, it is evident that attending Søndre Land school offsets—at least to some degree—the typical decline in PA levels. This further supports the conclusion that the Søndre Land FYSAK model contributes to maintaining pupils' levels of PA during this formative period. Given the social gradient in PA among youth (34) and the SES profile of the Søndre Land school (a larger proportion of students from lower SES backgrounds), this is also worthy of note. Pupils from lower SES backgrounds are less likely to access opportunities for PA through family and friends and thus engage in fewer sporting activities compared to their mid- and upper-middle-class peers (37). Thus, schools that provide PA opportunities during the school day are particularly important for pupils from lower SES groups and have the potential to contribute to narrowing social inequalities in PA. The importance of the school setting also applies to those pupils who do not have the opportunity for active travel to school. At Søndre Land school, a lower proportion of pupils were able to bike or walk to school compared to control schools, underscoring the contribution a FYSAK school can make to PA in such circumstances.

Overall, our results are indicative of a FYSAK school effect, which can be explained in terms of the sustained embedding of PA into pupils' daily routines over a three-year period. Multi-component whole-of-school approaches have been shown to have limited effectiveness, in part because of poor implementation (1, 38) and limited sustainability beyond the intervention period (39), One factor that has been identified as important for the introduction and embedding of such health promotion initiatives is proactive leadership (40) and supportive management practices such as resource allocation (41, 42). In the absence of such leadership whole school initiatives suffer from many of the same limitations as less comprehensive, short-duration interventions. Although this study cannot shed any light on these factors, information collected at the first visit to the FYSAK school identified the key role of the head teacher in developing and implementing the model.

Jago et al. (8) advocate for a much stronger focus on transforming the school context to support PA. Recent research on school improvement initiatives (including those relating to the health promoting school), has further supported the need to conceptualize schools as complex adaptive education systems if sustainability and implementation are to be adequately addressed (43–45). While there have been some recent initiatives underpinned by such a conceptualization (see for example, Creating Active Schools programme in Bradford, UK: 46)), our study has contributed knowledge to this type of organizational model. Furthermore, whole school approaches may well be too complex for schools to implement comprehensively, especially without considerable resources and specialist support. Nonetheless, a simplified focus on transforming the school context to create a culture that supports PA may be a viable, realistic alternative (8, 11, 12, 39). The FYSAK model illustrates how relatively small, systemic organizational adjustments can be integrated into everyday school life. As such, it appears to be a sustainable model that negates the need for any “intervention” and, consequently, is not only effective in increasing PA levels but also viable and equitable. Thus, the model contributes to PA becoming routinized during a formative period of the life course, supporting pupils in developing a physically active habitus: that is to say having a predisposition to move (47). In this regard, we think there is merit in moving beyond a focus on recommendations in terms of MVPA alone, notwithstanding its undeniable health benefits, to incorporate PA to break up and reduce sedentary time alongside incorporating lower intensity PA activities to generate fun and enjoyment. The FYSAK model incorporates relatively short, diverse physical activities that include all forms of movement and intensities, including low intensity activities such as walking, which create opportunities for more socially inclusive and engaging experiences for students. This way of developing a physically active habitus is more likely to foster lifelong engagement in PA [see., e.g., (48)].

### 4.1 Limitations

Notwithstanding the high participation rate at Søndre Land school, we recognize the limitations of our analyses in terms of its relatively small sample size, which limited our ability to control for SES differences between the two school categories. However, given the higher proportions of pupils from lower SES backgrounds at Søndre Land school, it is likely that this might have diminished PA levels. In addition, some participants did not provide valid PA measurements for all years. Although analyses of mean PA strengthened the power of these analyses somewhat, the small sample inhibited in-depth analyses and inclusion of relevant covariates such as SES, body mass index and travel mode to/from school. Further, although accelerometry is widely regarded as the best way to assess habitual PA (29), it is not without its limitations, such as not being able to measure water activities. Furthermore, because of their placement at the hip, their ability to provide precise measurements of cycling, arm-intensive activities, and activities on a gradient are limited (49). The amount of time spent cycling or swimming was thus recorded in the questionnaire and the majority of participants reported zero hours of both activities. Consequently, this limitation seems unlikely to have led to an underestimation of the true activity level, at least to a large degree. In addition, there is still no consensus regarding processing criteria (50), which may influence the results and inhibit data comparison across studies using different criteria. However, the use of average daily cpm^−1^ as the measure of PA level removes some of the differences introduced by using different intensity-specific cut-points. Lastly, FAS III was used to measure SES. Although this version is considered to provide absolute and relative inequalities between groups of adolescents, both within and between countries (33), ceiling effects have been identified in highly affluent study samples, often found in Norway (32). The instrument is therefore regarded as only providing a rough measure of SES. It is important to note that these limitations apply to both Søndre Land school and control schools and will thus likely not influence the results.

### 4.2 Implications for school PA policy and practice

Notwithstanding these limitations, our findings raise important policy and practice issues about how schools can effectively, sustainably and equitably promote PA alongside their educational mandate. The ongoing interest in whole-of-school approaches—including schools as complex adaptive systems—reflects the view that they are more likely to ensure that health promotion becomes institutionalized in the everyday structures and processes of the school (51) and thus more likely to ensure effective, equitable and sustainable ways of promoting health and PA (38, 52). Both frameworks, however, introduce complexity into the everyday reality of schools, an unintended consequence of which may be that they are less likely to be successfully adapted, implemented, and sustained. As far as PA promotion is concerned, our results suggest an alternative way forward. The FYSAK model has embedded PA into the fabric of the school's daily routines through a relatively straightforward shift in the way time is used. The model was initiated, developed and led by the headteacher and implemented by all teachers. Educational leadership has been shown to be influential in stimulating cultural change, which has been widely recognised as important in anchoring and sustaining health promotion initiatives (41, 42). Research on leadership in health promotion is sparse, especially with regard to PA promotion. Nonetheless, the extant research indicates that it is likely that leadership played an influential role in shifting the school culture towards that of valuing PA as well as providing resources to ensure its sustainability (40). Given the competing pressures lower secondary schools especially face, such leadership is particularly likely to be important and warrants further research in Norway as elsewhere.

In terms of inclusion, the FYSAK model is an example of a school population approach to PA promotion that reaches all students, including those most disadvantaged. Thus, it is a model that has the potential to minimize social inequalities in health in general and PA in particular (53). Given the widespread acceptance that the so-called standard school does not exist (10), it is also a model that can be adapted to each school's location, resources and circumstances. In this regard, the model is widely applicable to other schools in Norway and beyond. However, while schools can be important settings for health promotion, their potential effect is likely to be marginal if they are not supported by actions in other domains. Policies that give every child the “best start in life” (54)—in this case with regard to developing a physically active habitus—during the formative years of preschool and primary school are equally important. These early years are crucial for socializing children into viewing movement and PA as integral parts of everyday life.

Finally, it is worth noting that if schools are to address equity thereby narrowing social inequalities in health and PA, then a re-distribution of resources is required towards disadvantaged communities. Schools situated in areas of disadvantage often face unique difficulties with regard to resource limitations and competing academic demands. If the aim is to ensure equitable opportunities for all pupils, then policymakers would be advised to allocate targeted funding and tailored support to under-resourced schools. This not only enables these schools to implement effective PA initiatives, it also helps bridge the gap in health outcomes between socio-economic groups, including in relation to PA.

## 5 Conclusion

Our results suggest that if schools are to become settings for sustainable PA promotion, then a shift away from top-down, limited-duration interventions that leave little organizational imprint once completed is necessary. In the same vein, we also raise questions about the continuing advocacy of complex whole-of-school models because they may well overwhelm schools, especially lower secondary schools. On the basis of our results, we conclude that the FYSAK model offers a framework for systematically providing realistic opportunities for being physically active during the school day if it becomes seamlessly integrated—in other words institutionalized—into schools' daily practice of teaching and learning. The model offers a way of balancing academic priorities with the desirability of developing active lifestyles. The prerequisites for such a model are likely to be an empowering school leadership, engaging teachers in collaborative processes, and facilitating pupil involvement (40). These are, however, our tentative conclusions and require further research. In addition, future research should explore whether the FYSAK model influences sports participation outside of school.

## Data Availability

The raw data supporting the conclusions of this article will be made available by the authors, without undue reservation.

## Footnotes

^1^A Norwegian term derived from *fysisk aktivitet* [physical activity], referring to a structured, school-based system for promoting daily physical activity.

^2^ https://www.sondre-land.kommune.no/enheter/skolene-i-sondre-land/sondre-land-ungdomsskole/
